# Human genetic variant E756del in the ion channel *PIEZO1* not associated with protection from severe malaria in a large Ghanaian study

**DOI:** 10.1038/s10038-021-00958-2

**Published:** 2021-07-07

**Authors:** Thorsten Thye, Jennifer A. Evans, Gerd Ruge, Wibke Loag, Daniel Ansong, Tsiri Agbenyega, Rolf D. Horstmann, Jürgen May, Kathrin Schuldt

**Affiliations:** 1 Department of Infectious Disease Epidemiology, Bernhard Nocht Institute for Tropical Medicine, Hamburg, Germany; 2 Department of Molecular Medicine, Bernhard Nocht Institute for Tropical Medicine, Hamburg, Germany; 3 Kumasi Centre for Collaborative Research in Tropical Medicine, Kumasi, Ghana; 4 University of Science and Technology, School of Medical Sciences, Kumasi, Ghana, Departments of Child Health and Medicine, Komfo Anokye Teaching Hospital, Kumasi, Ghana; 5 German Center for Infection Research (DZIF), Hamburg-Lübeck-Borstel-Riems, Germany

**Keywords:** Malaria, Genetic association study

## Abstract

Recently, a common genetic variant E756del in the human gene *PIEZO1* was associated with protection from severe malaria. Here, we performed a genetic association study of this gain-of-function variant in a large case-control study including 4149 children from the Ashanti Region in Ghana, West Africa. The statistical analysis did not indicate an association with protection from severe malaria and, thus, providing evidence against a strong protective effect of the *PIEZO1* E756del variant on severe malaria susceptibility.

## Communication

First evidence for a role of the human *PIEZO1* variant E756 (rs59446030) in infections with *Plasmodium falciparum* was provided by Ma et al. [1]. Following that publication, we analyzed the common E756 variant in a large case-control study (*n* = 4149) on severe malaria (SM) in the Ashanti Region of Ghana, including 2413 SM cases and 1736 unaffected controls. This study group has proven to be a valuable resource in the past [2]. Among others, it has been used to successfully delineate the effects of established hemoglobin variants on SM subphenotypes, and it was the first study to identify a variant in the human gene *ATP2B4* influencing the susceptibility to SM, which subsequently has been replicated in several other studies [2–4].

Genotyping of the *PIEZO1* variant E756 (rs59446030) in the Ghanaian study group was done by allele-specific hybridization with fluorescence resonance energy transfer (FRET) in a LightCycler^®^ 480 device [5, 6] for all study participants (*n* = 4149) (Suppl. Table 1). The genotyping revealed the presence of the Wildtype (WT), a E756 deletion (TCC_del_, E756del) and, at a lower frequency, an insertion (TCC_ins_, E756ins) (Table 1). For a subset of the DNA samples (*n* = 10) the three different *PIEZO1* alleles identified in our study group were validated by Sanger sequencing (Supplementary Fig. 1). Allele frequencies of E756del in the Ghanaian cases and controls were 0.19 and 0.20, respectively, at a level similar to that of other populations of African descent [1]. Recently, Nguetse et al. described an association of the same mutation E756del in the human gene *PIEZO1* with protection against severe malaria in a Gabonese case-control study [7]. The authors postulate that their results support the notion of *PIEZO1* being an important host susceptibility factor for falciparum malaria. In contrast to the findings by Nguetse et al., the statistical analysis of the E756del variant did not indicate an association with protection from SM in our large Ghanaian study group.

**Table 1:** Association analyses of *PIEZO1* E756del (rs59446030) with severe malaria in the Ghanaian case-control study

	SM study group^a^ *n*_total_ = 4149		
	SM cases *n* = 2413 (%)	Controls *n* = 1736 (%)	Odds ratio^b^, 95% CI, *p* value
*PIEZO1* E756 genotype			
WT/WT	1483 (61.5)	1037 (59.8)	Ref
WT/del	699 (29.0)	523 (30.1)	0.91 (0.79–1.05) *p* = 0.19
del/del	91 (3.8)	85 (4.9)	0.71 (0.52–0.98) *p* = 0.04
WT/ins	107 (4.4)	70 (4.0)	1.04 (0.75–1.42) *p* = 0.83
del/ins	25 (1.0)	16 (0.9)	1.17 (0.61–2.24) *p* = 0.63
ins/ins	8 (0.3)	5 (0.3)	1.26 (0.40–3.94) *p* = 0.70
Hemoglobin type			
HbAA	2128 (88.4)	1,314 (75.8)	Ref
HbAS	34 (1.4)	263 (15.2)	0.08 (0.05–0.12) *p* < 0.00001
HbAC	239 (9.9)	148 (8.5)	0.34 (0.12–0.99) *p* = 0.05
HbCC	6 (0.3)	9 (0.5)	0.93 (0.75–1.17) *p* = 0.55
Median age, months (range)	20 (7–117)	30 (7–116)	
Sex (female, %)	46.1	47.1	
Ethnic group, *n* (%)			
Akan	1605 (66.5)	1260 (72.6)	
Northerner	698 (28.9)	442 (25.4)	
Ewe	73 (3.1)	24 (1.4)	
Ga	37 (1.5)	10 (0.6)	

*SM* severe malaria, *CI* confidence interval.

^a^Severe malaria case group includes discrete and partly overlapping phenotypes, severe malaria anemia defined as hemoglobin level <5 g/dl, cerebral malaria defined as Blantyre coma score <3, and hyperlactatemia; prostration, hyperparasitemia, respiratory distress.

^b^Results of logistic regression adjusted for age, sex and ethnic group.

We applied the logistic regression model as described by Nguetse et al., which tests for the effect of the heterozygous E756del genotype (WT/del) on SM compared to the homozygous E756 genotype (WT/WT), and we adjusted for sex, age, ethnicity, and HbAS status. The model yielded an odds ratio (OR) of 0.91 (95% confidence interval, CI, 0.79−1.05, *p* = 0.23; Table 1). Similarly, there was no evidence for an effect of the other E756del/ins genotypes on the risk of developing SM (Table 1), neither did we observe an interaction of E756 genotypes with hemoglobin types (HbS, HbC). Weak evidence for an association with protection from SM was observed for the E756del/del genotype (OR 0,71, 95% CI 0.52–0.98, *p* = 0.04; Table 1). However, this result should be interpreted with caution, because it is based on a relatively small difference in frequency of genotypes between cases and controls (1.1%) and moreover the *p* value would not be below a significance level of (*p* = 0.05) after corrections due to multiple testing.

Discrepancies between the studies may be the result of inherent geographical differences, i.e., the degree of malaria exposure, or different genetic backgrounds of ethnic groups, or sample size (Gabonese study *n*_total_ = 446; Ghanaian study *n*_total_ = 4149). In addition, in their study Nguetse et al. included mild malaria cases as controls, whereas the control group of the Ghanaian study consisted of apparently healthy children at time of recruitment [2]. As there is considerable heterogeneity of malaria-associated loci in terms of mode of effect as well as across populations and subphenotypes [8], it is important to note, that a failed replication of a marker-trait association could also indicate a population-specific effect.

In conclusion, our results provide evidence against a strong protective genetic effect of the *PIEZO1* E756del variant on SM susceptibility and underline the need for further studies in populations exposed to falciparum malaria.

## Supplementary information

FRET_primer

Piezo1 E756del melting curves

## Data Availability

Genotypes generated in this study can be retrieved from the data sharing platform ZENODO (https://zenodo.org; 10.5281/zenodo.4925969).
