# Supplementary material for: Human genetic variant E756del in the ion channel PIEZO1 not associated with protection from severe malaria in a large Ghanaian study
Source: J Hum Genet. 2021 Jul 7;67(1):65–7. doi: 10.1038/s10038-021-00958-2 (PMC8727285; doi:10.1038/s10038-021-00958-2)
Supplement: Supplementary file 1 — FRET_primer [file 10038_2021_958_MOESM1_ESM.docx]

Supplementary Table1

Forward

5’-GGACATGGCACAGCAGACTGG-3’

Reverse

5’-CAGGCAGGATGCAGTGAGTGG-3’

Anchor

5’-GCCACGCCCAGCCCCTCGTCCCTGG- fluorescein

Sensor

5’-cyanine 5-GTCCTCCTCCTCCTCCTCCTCCTCCTG-phosphate

Primers, anchor and sensor applied for FRET based genotyping of the Piezo1 E756del variant.
