# Supplementary figures and images for: Human genetic variant E756del in the ion channel PIEZO1 not associated with protection from severe malaria in a large Ghanaian study
Source: J Hum Genet. 2021 Jul 7;67(1):65–7. doi: 10.1038/s10038-021-00958-2 (PMC8727285; doi:10.1038/s10038-021-00958-2)

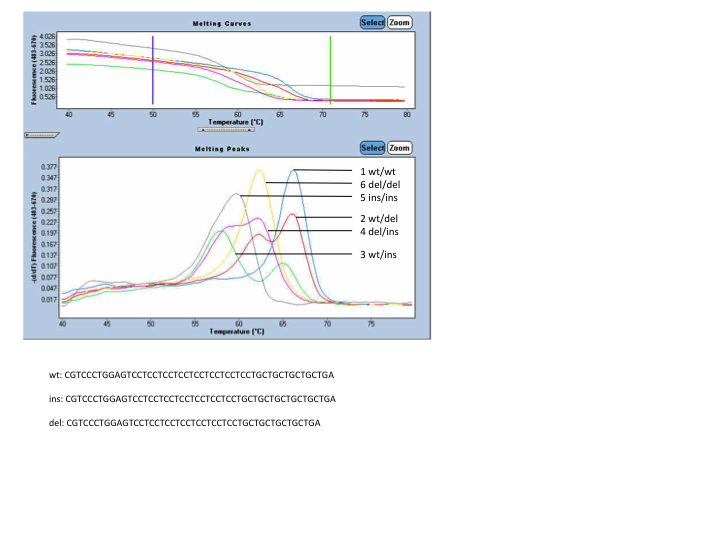

Supplement: Supplementary file 2 — Piezo1 E756del melting curves [file 10038_2021_958_MOESM2_ESM.tif]
